# Fetus-in-fetu: mimicking teratoma on antenatal ultrasound

**DOI:** 10.1515/crpm-2022-0024

**Published:** 2023-03-10

**Authors:** Ines Mazhoud, Wissal Skhiri, Chiraz Hafsa, Amel Maghrebi, Amine Ksiaa, Mohamed Maatouk, Amina Ben Salem

**Affiliations:** Department of Radiology, Maternity Center, Monastir, Tunisia; Department of Pediatric Surgery, Fattouma Bourguiba Monastir, Monastir, Tunisia; Department of Surgery, Fattouma Bourguiba Monastir, Monastir, Tunisia; Department of Radiology, Fattouma Bourguiba Monastir, Monastir, Tunisia

**Keywords:** fetus in fetu, newborn, radiologic findings

## Abstract

**Objectives:**

Fetus-in-fetu is a rare congenital anomaly that occur secondary to abnormal embryogenesis in a diamniotic monochorionic pregnancy. Its diagnosis can be accurately made by imaging ultrasonography, radiography, computed tomography, or magnetic resonance imaging. Differential diagnosis is an important issue because FIF, teratoma and cystic meconium peritonitis are very different in terms of their respective disease courses.

**Case presentation:**

This is an interesting rare case of a 22-year-old pregnancy woman, presented for a routine antenatal ultrasound. The diagnosis of a fetus-in-fetu was suspected, complete surgical excision of the lesion was performed and the diagnosis was histopathologically confirmed

**Conclusions:**

We describe also the common characteristic of FIF as revealed by prenatal and postnatal US, postnatal MRI, and the operative findings.

## Introduction

Fetus-in-fetu (FIF) is a rare congenital anomaly that occur secondary to abnormal embryogenesis in a diamniotic monochorionic pregnancy [1]. In the past, the level of preoperative FIF diagnosis was low, but this has improved recently due to improvements in the technical skill of ultrasonography (US) and increased interest in prenatal examinations. In particular, MRI have greatly assisted accurate diagnosis. Differential diagnosis is an important issue because FIF, teratoma and cystic meconium peritonitis are very different in terms of their respective disease courses [2]. This is an interesting rare case of a 22-year-old pregnancy woman, who presented for routine antenatal ultrasound. We describe the common characteristic of FIF as revealed by prenatal US, postnatal US, postnatal MRI, and the operative gross findings. The diagnosis of a fetus-in-fetu was made, complete surgical excision of the lesion was performed and the diagnosis was confirmed histopathologically.

## Case presentation

**Figure 1:**
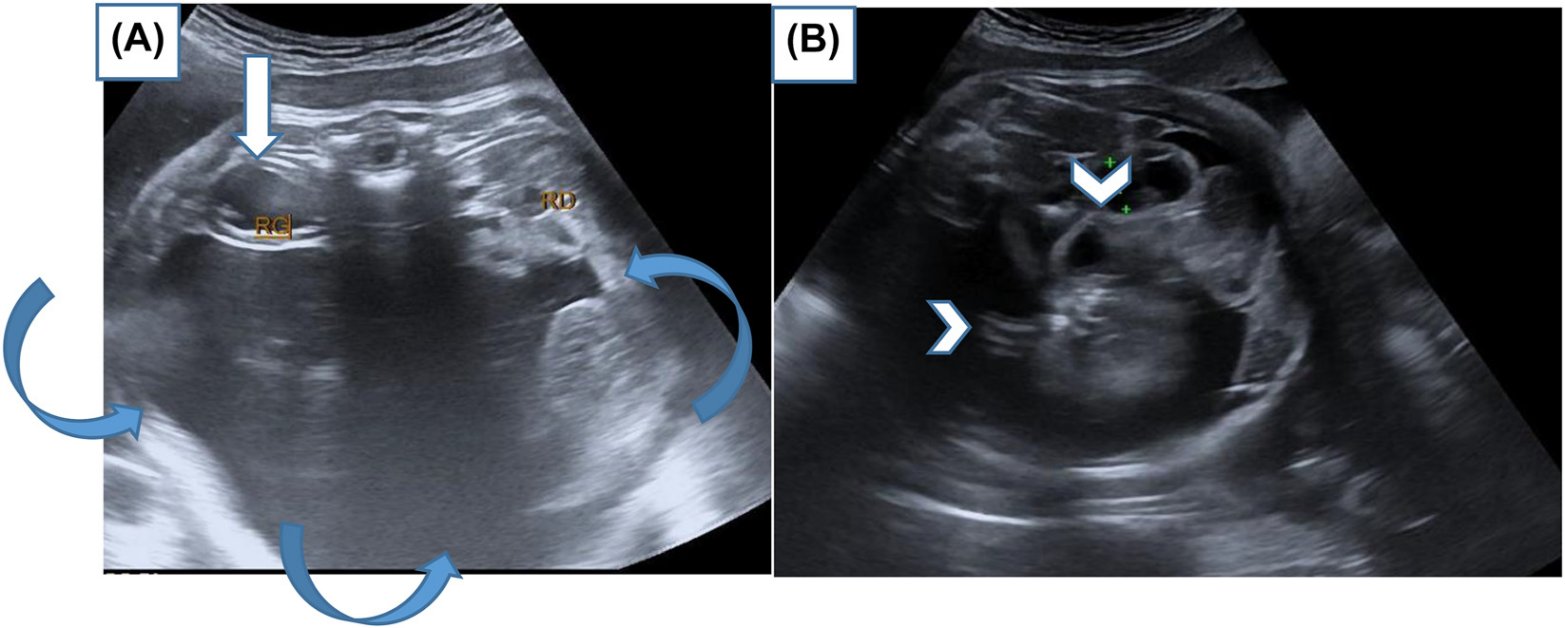
(A, B): Antenatal ultrasound images show heterogeneously echogenic intra-abdominal mass, in the low abdomen of the foetu, with multiple calcifications (arrow head), surrounded by a fluid-filled sac (curved arrows), in the fetal abdomen. The right kidney was compressed pushed posteriorly by the mass (arrow).

**Figure 2:**
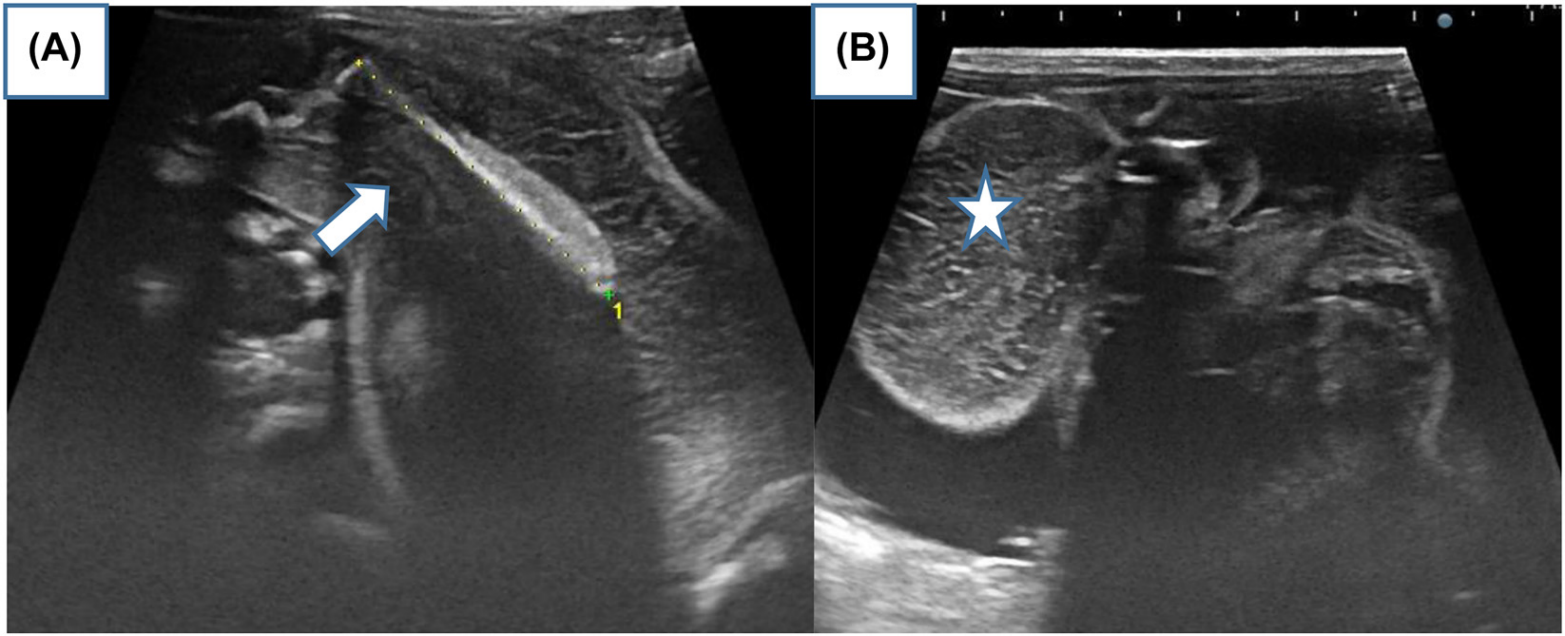
(A, B): Postnatal ultrasound of the newborn shows show heterogeneously echogenic mass with linear echogenic structure resembling bone (arrow) and intestines (star).

**Figure 3:**
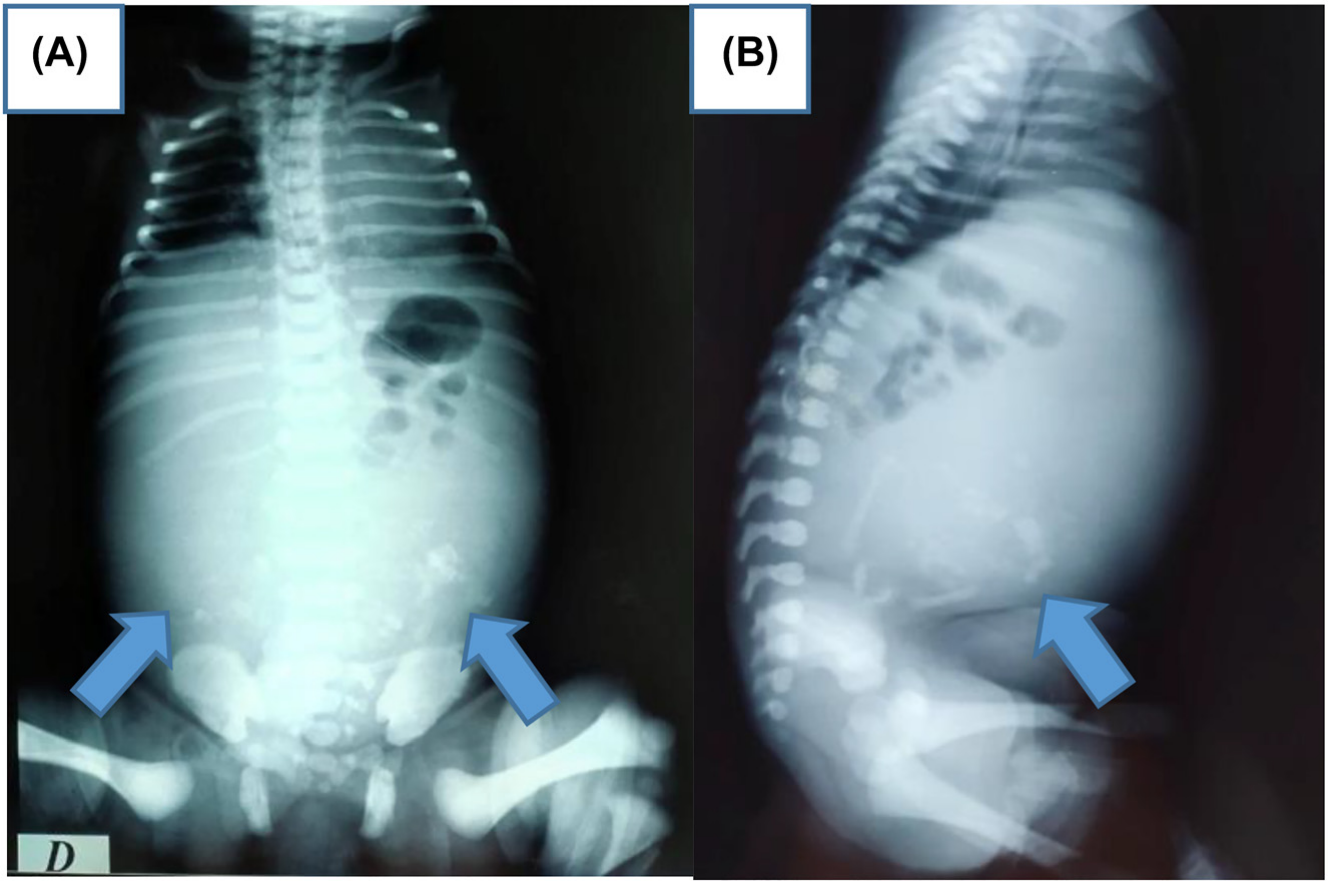
(A, B): Radiograph showing fetus skeleton inside the abdomen of the newborn resembling well-formed limb bones and rib (arrows).

**Figure 4:**
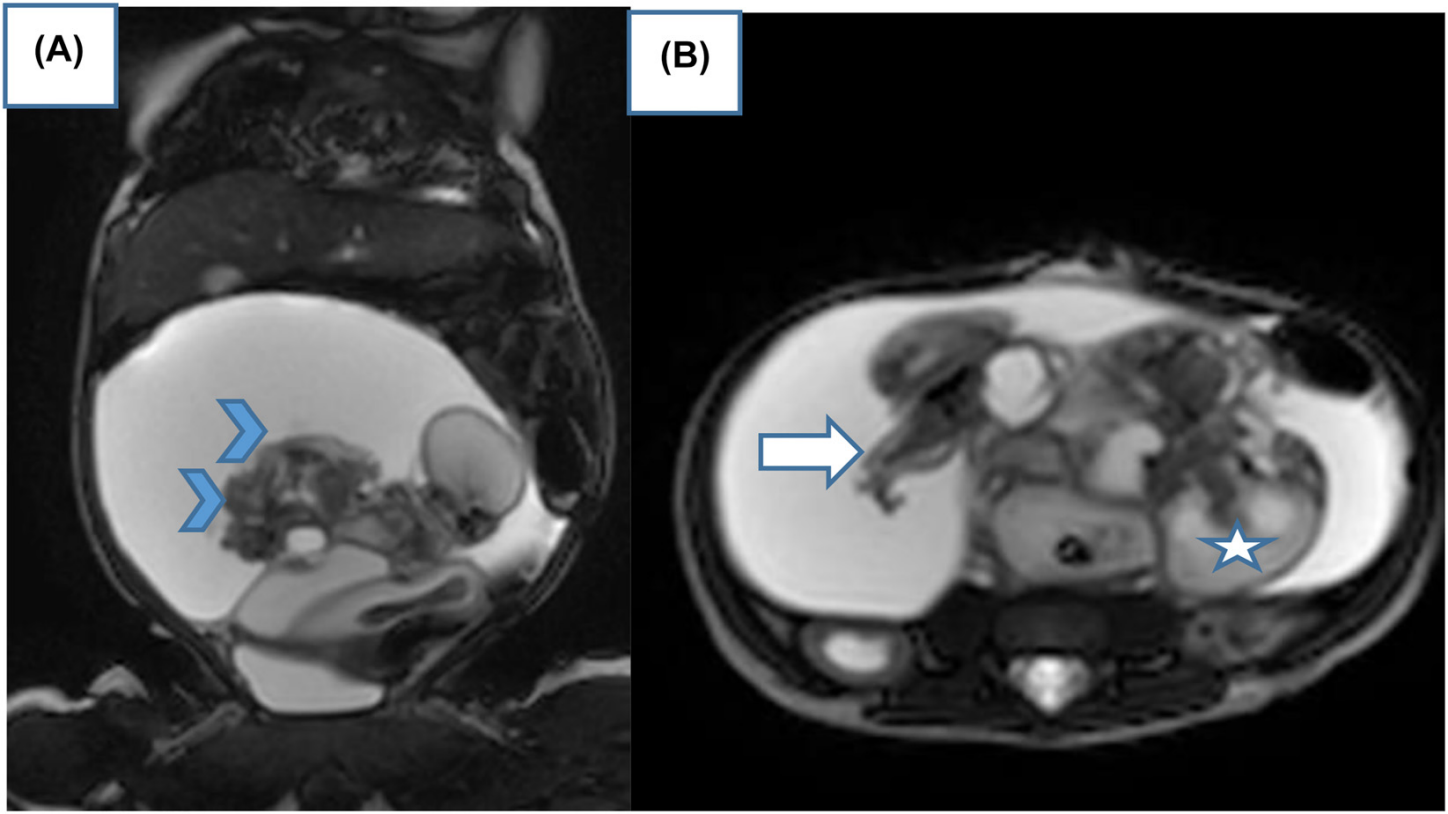
(A, B): Postnatal MRI shows the same feature of US. Heterogeneously cystic mass with calcifications resembling limbs (arrow). It is also contains structures resembling intestines (head arrow) and brain (star).

**Figure 5:**
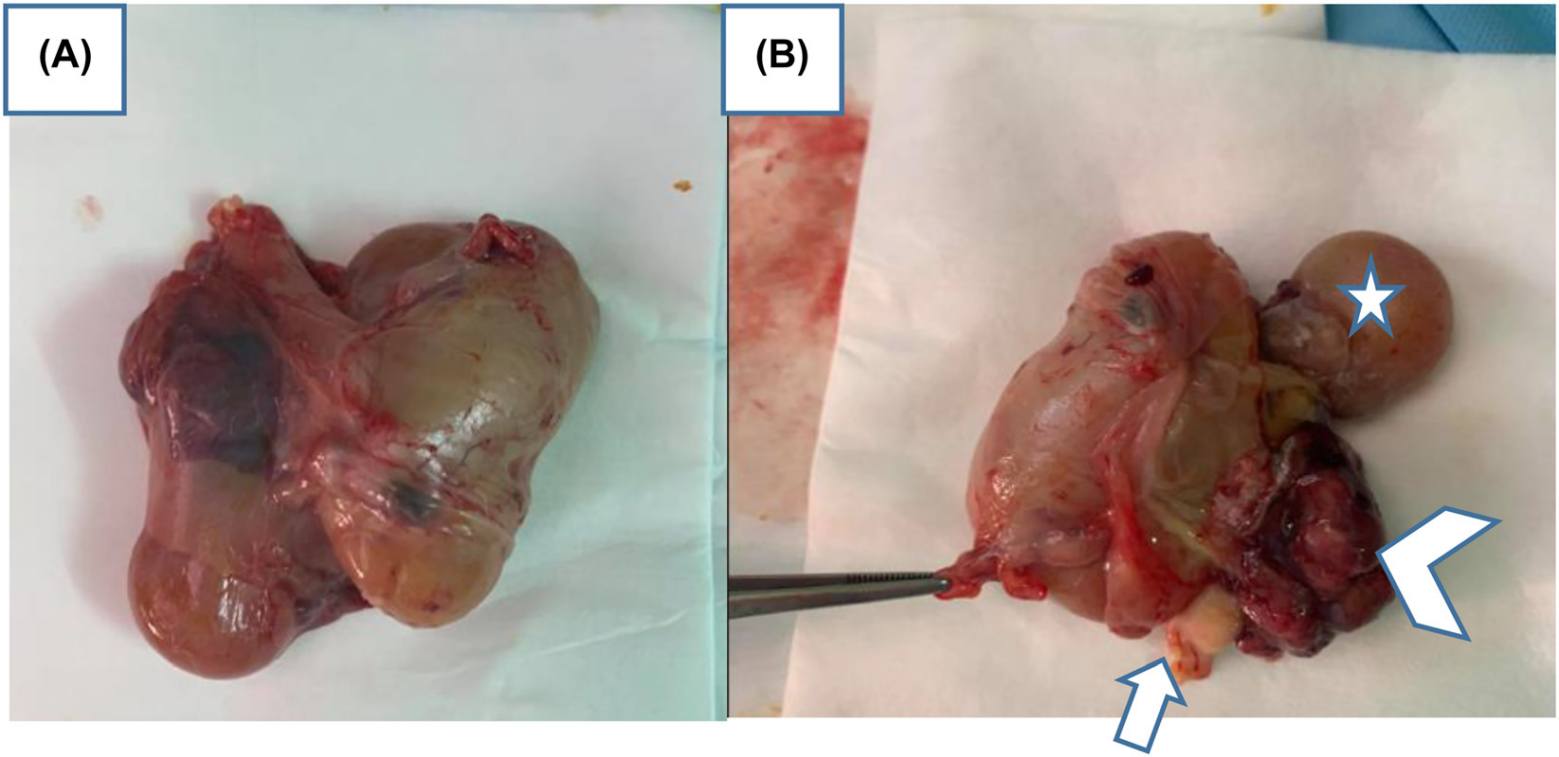
(A, B). In the operative findings, encapsulated fetus dissecting out of the abdomen of the newborn was found. (A) The solid portion (B) has a number of rudimentary limb buds (head arrow), vascular structures, intestines (arrow) and brain (star).

An ultrasound for 22-year old female G_1_P_1_, performed in the 35^TH^ SA of pregnancy, showed intrauterine viable fetus. The family history was negative for congenital malformations, and there was no history of medication and drug use during pregnancy. Antenatal ultrasound images show heterogeneously echogenic intra-abdominal mass, in the low abdomen of the fetus, with multiple calcifications, surrounded by a fluid-filled sac, in the fetal abdomen. The right kidney was compressed pushed posteriorly by the mass. The biometry measurements were appropriate for gestation age (Figure 1). No other congenital anomaly was detected in the fetus. The provisional diagnosis of a teratoma was made and the patient was advised second ultrasound of the newborn after delivery. The patient delivered a female baby at 39 weeks of gestation. Ultrasound of the infant was done on the same day of delivery. It showed a large complex mass measuring 10 × 6 cm in the fetal abdomen. The right kidney was compressed pushed posteriorly by the mass. Osseous elements resembling limb bones and intestines were seen in the mass (Figure 2). The diagnosis of fetus-in-fetu was made with the second possibility of teratoma. We completed by X-ray which showed fetus skeleton inside the abdomen of the newborn resembling well-formed limb bones and rib (Figure 3) which confirmed the diagnosis of fetus-in-fetu. Postnatal MRI shows the same feature of US (Figure 4). Heterogeneously cystic intra-abdominal mass with calcifications resembling limbs. It is also contains structures resembling intestines and brain. On subsequent surgery, the mass was present in intraperitnoeum. It was well encapsulated. It was removed in toto. In the operative findings, encapsulated fetu dissecting out of the abdomen of the newborn was found (Figure 5). On examination, it was a malformed baby weighing approximately 450 g, the solid portion has a number of rudimentary limb buds, vascular structures, intestines and brain. In our case, we completely resected the mass with no subsequent complication to the best of our knowledge. It is necessary to keep the child in follow‐up and surveillance.

## Discussion

Fetus-in-fetu is rare developmental abnormality with less than 200 cases reported in the literature [1]. It is secondary to the abnormal embryogenesis in a diamniotic, monochorionic pregnancy. There is a slight male predominance [2] and 89% of FIF lesions were noted before 18 months of ages [3]. Studies of genetic markers, such as blood group, sex chromosome constitution, protein polymorphisms, and DNA marker, suggested that host infants and their fetiform mass are genetically identical [1]. FIF usually presents as a foetiform osseous mass, often in the abdomen of its host, with the retroperitoneum being the most common site to be affected (almost 80%) [2]; however extremely rare in the pelvis, scrotal sac, sacrococcygeal region, mesentery, right iliac fossa and cranial cavity [2]. In contrast, a teratoma is most commonly found in the sacrococcygeal, gonadal, mediastinal, and CNS. In cystic meconium peritonitis, the most common site of occurrence is intra-abdominal followed by the hollow viscus. Different organs can be seen in FIF, including vertebral column, limbs, central nervous system, gastrointestinal tract, vessels, genitourinary tract and heart [1, 4]. FIF is usually within a capsule, which is an amnion-like membrane and It is supplied by a single feeding vessel [4, 5]. Absence of an independent circulatory system explains the subsequent growth retardation [5]; the mass may compress the surrounding organs and tissue and causes complications such as maldevelopment of surrounding organs [4] and death for the host. Prenatal ultrasound is a useful tool to identify developed organs in the FIF, evaluate the size and spatial relationship of the mass, it is seen as a mass with a complex echo pattern, with fluid, soft tissue and calcification all being often identified within it. Fetus-in-fetu should be considered in the differential diagnosis of any calcified intra-abdominal mass seen on antenatal ultrasound [6, 7]. In our case, unfortunately, we only thought of the teratoma as a prenatal diagnostic. The presence of well differentiated organogenesis is the key feature to facilitates differential diagnosis with teratomas, also the detection of fetal heart beat [4, 7]. FIF and teratoma have similar sonographic features on ultrasound examination, and hence, the risk of misdiagnosis is present. To facilitate the distinction, in FIF, the mass was divided into two parts, the peripheral fluid-filled cystic portion and the central solid portion “floating” within [7]. The cystic portion was not divided by a septum. A teratoma is different in that it is often a multi-loculated cystic mass, or a mixed mass of solid and cystic portion without clear border [7]. In cases of cystic meconium peritonitis, free air or ascites may be observed. Very few cases have been diagnosed prenatally using sonogram and the most cases were detected in 2nd and 3rd trimester, but earlier case was demonstrated at 16 weeks’ gestation [4]. US can help also to guide the ex utero intrapartum treatment procedure for newborn FIF, or offer the reasonable option of early termination for severe cases. Computed tomography scan can give a more accurate diagnosis and defines the relation of the FIF with the other intraabdominal structures. In addition, CT three-dimensional reconstruction can completely display the axial bone system and the limbs in the FIF, which are core in the diagnosis as pointed out by Willis and Lewis [5]. It facilitates also the distinction between calcification of cases of FIF from those of a teratoma or cystic meconium peritonitis. In contrast with the bony calcification of FIF, the calcified features of teratoma have more of a tooth-like appearance, Whereas those of cystic meconium peritonitis are amorphous, deposited in a “peripheralized” capsule. In recent years, MRI has also been used to diagnose FIF, which can clearly identify the soft tissues and organs surrounding the FIF, thereby providing valuable imaging data for the formulation of surgical strategies. Compared with CT, MRI is an ideal imaging modality which avoids the need for iodine contrast and eliminates the risk of ionizing radiation, it is considered safe in pregnancy. The role of tumor markers is confined to the differentiation between FIF and other causes of intra-abdominal calcification, including teratoma, neuroblastoma, adrenal hemorrhage, meconium pseudocyst, and viral infection. The most used markers are β-human choriogonadotropin (hCG), AFP, and urine homovanillic acid [2]. Maternal and host serum alpha fetoprotein levels may also be raised [5]. After birth, plain radiography may reveal bone structures; while failure to detect such structures does not rule out the diagnosis, it is detected only on 50% [5]. CT offers a detailed view of the structures that compose the mass, its vascular anatomy, and its relationship with the surrounding organs, important information for surgical management. Magnetic resonance imaging is gaining importance due to its high tissue contrast and spatial resolution. Consistent with the theory of Willis, in our case, the limbs was detected by the X-ray. It was radio‐opaque, the ultrasound showed an intraperitoneal, heterogenous mass and MRI confirmed the diagnosis. Treatment of fetus in fetu is essentially surgical and excision gives complete recovery. Despite being a benign condition, and complete surgical excision is curative, the potential of malignancy should be kept in mind. Till now, there is only one case reported in literature with malignant transformation [8]. Follow-up with imaging and tumor markers is advised in cases showing immature components.

## Conclusions

Fetus-in-fetu is a rare clinical entity that occurs in diamniotic and monochorionic pregnancy and results from error in embryogenesis. Its diagnosis can be accurately made by imaging ultrasonography, plain radiography, computed tomography, or magnetic resonance imaging. Fetus-in-fetu should be considered in the differential diagnosis of any calcified intra-abdominal mass seen on antenatal or postnatal ultrasound The main differential diagnosis is Teratoma.
